# Supplementary material for: MDA5 disease variant M854K prevents ATP-dependent structural discrimination of viral and cellular RNA
Source: Nat Commun. 2021 Nov 18;12:6668. doi: 10.1038/s41467-021-27062-5 (PMC8602431; doi:10.1038/s41467-021-27062-5)
Supplement: Supplementary file 1 — Supplementary Information [file 41467_2021_27062_MOESM1_ESM.pdf]

MDA5 autoimmune disease variant M854K prevents ATP-dependent structural discrimination of viral and cellular RNA

Yu, Herrero del Valle, Singh *et al.*

Supplementary Information

Supplementary Figures 1 to 8

Supplementary Figures

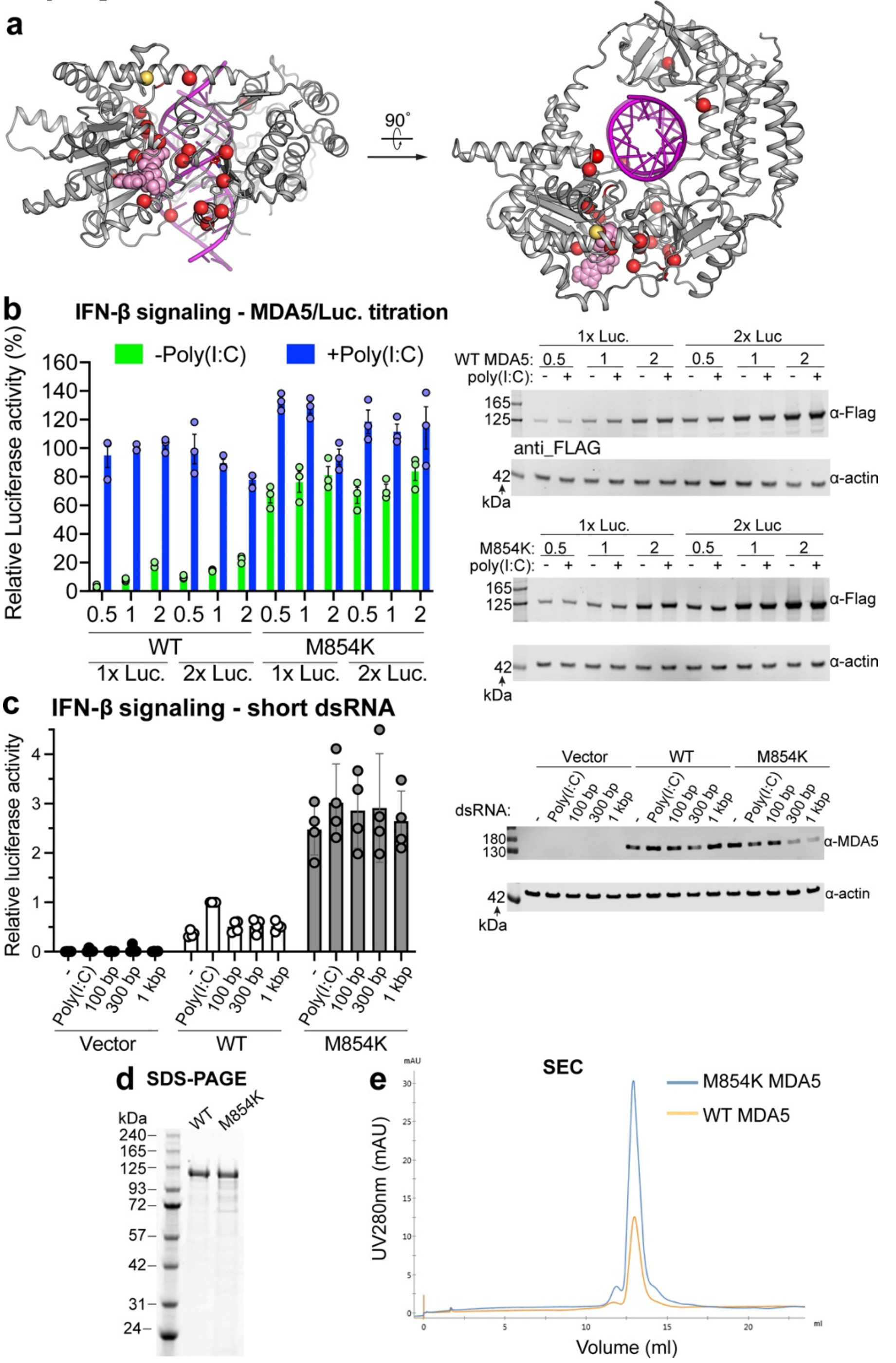

Supplementary Fig. 1. IFN-β signaling assay and size-exclusion chromatogram of M854K MDA5. (**a**) WT MDA5 with ADP-AlF4 bound showing the location of M854K (yellow) and selected other reported disease-associated mutations (red; residues 331, 393, 444, 449, 452, 489, 583, 720, 773, 779, 781, 802, 822, 824, 848, 979). Pink, ADP-AlF4; magenta, dsRNA. (**b**) IFN-β assay in HEK293T cells with titration of both MDA5 and luciferase plasmids. Luciferase activity was normalized against WT MDA5 +Poly(I:C). The fold of MDA5 plasmid (0.5, 1 or 2) and of luciferase plasmid (Luc.; 1 or 2) is indicated. Error bars show the mean ± SEM; n = 3 distinct samples. Right panel: Western blots showing the MDA5 expression level. MDA5 was stained with an anti-FLAG antibody. (**c**) IFN-β reporter cell signaling assay in HEK293T cells stimulated with poly(I:C), with linear *in vitro*-transcribed dsRNAs of different lengths, or without dsRNA stimulus (“-“). Error bars show the mean ± standard deviation; n = 4 distinct samples. Right panel: Western blots showing the MDA5 expression level. MDA5 with an anti-MDA5 antibody. Signaling assays were repeated independently at least three times with similar results. (**d**) Coomassie-stained SDS-PAGE of purified MDA5 used for biochemical assays. (**e**) Size-exclusion chromatograms of WT and M854K MDA5 at the final purification step.

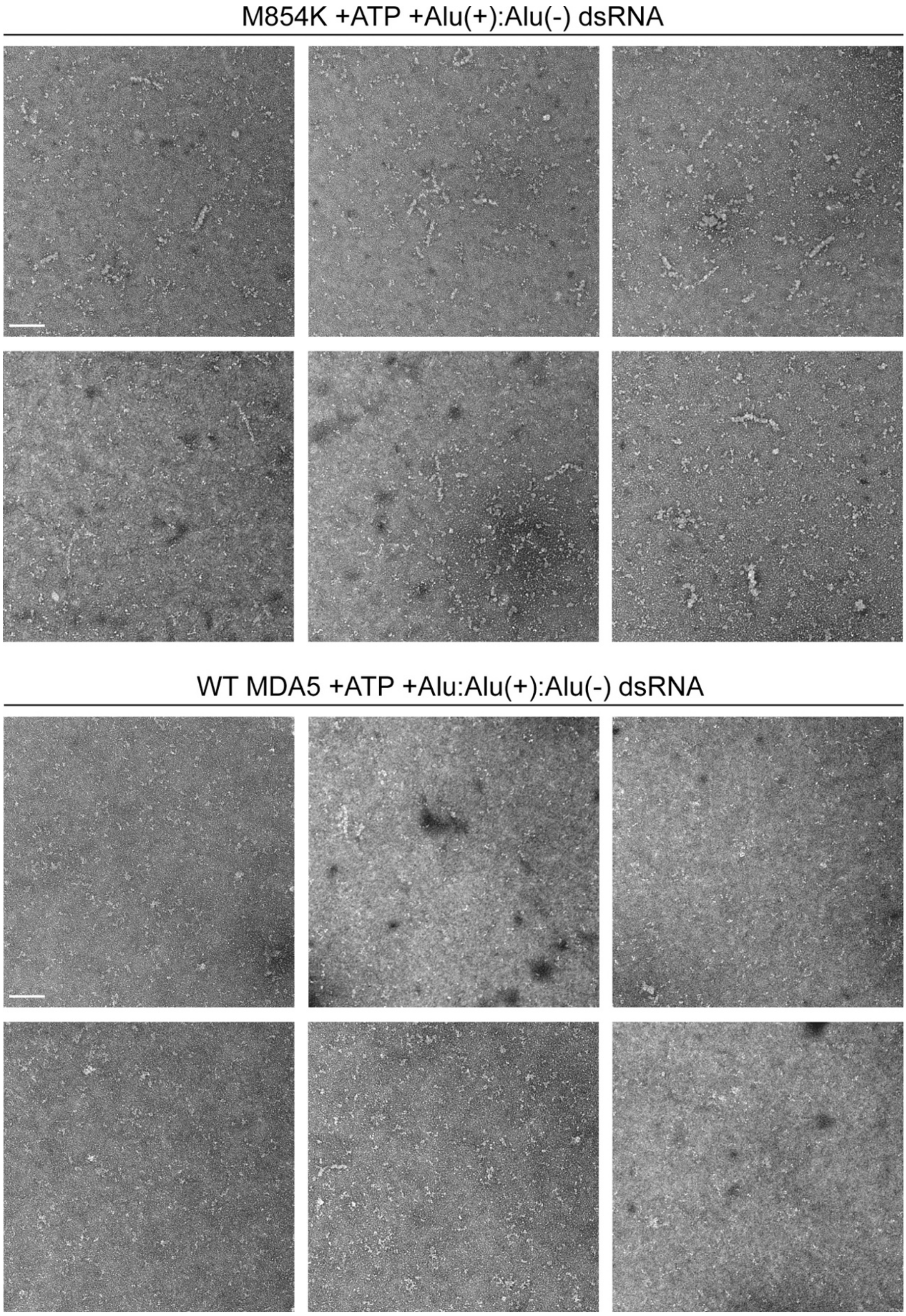

Supplementary Fig. 2. Filament formation of M854K MDA5 on Alu(+):Alu(-) dsRNA. Negative stain electron micrographs of M854K and WT MDA5 with Alu(+):Alu(-) dsRNA, in the presence of ATP. 22 filaments can be counted in the six micrographs with M854K MDA5. Two filaments are visible in the micrographs with WT MDA5 and Alu(+):Alu(-) dsRNA. No filaments were visible in the absence of dsRNA with both WT and M854K MDA5 (see **Fig. 2d**). Scale bars, 100 nm. Images from a duplicate experiment gave similar results.

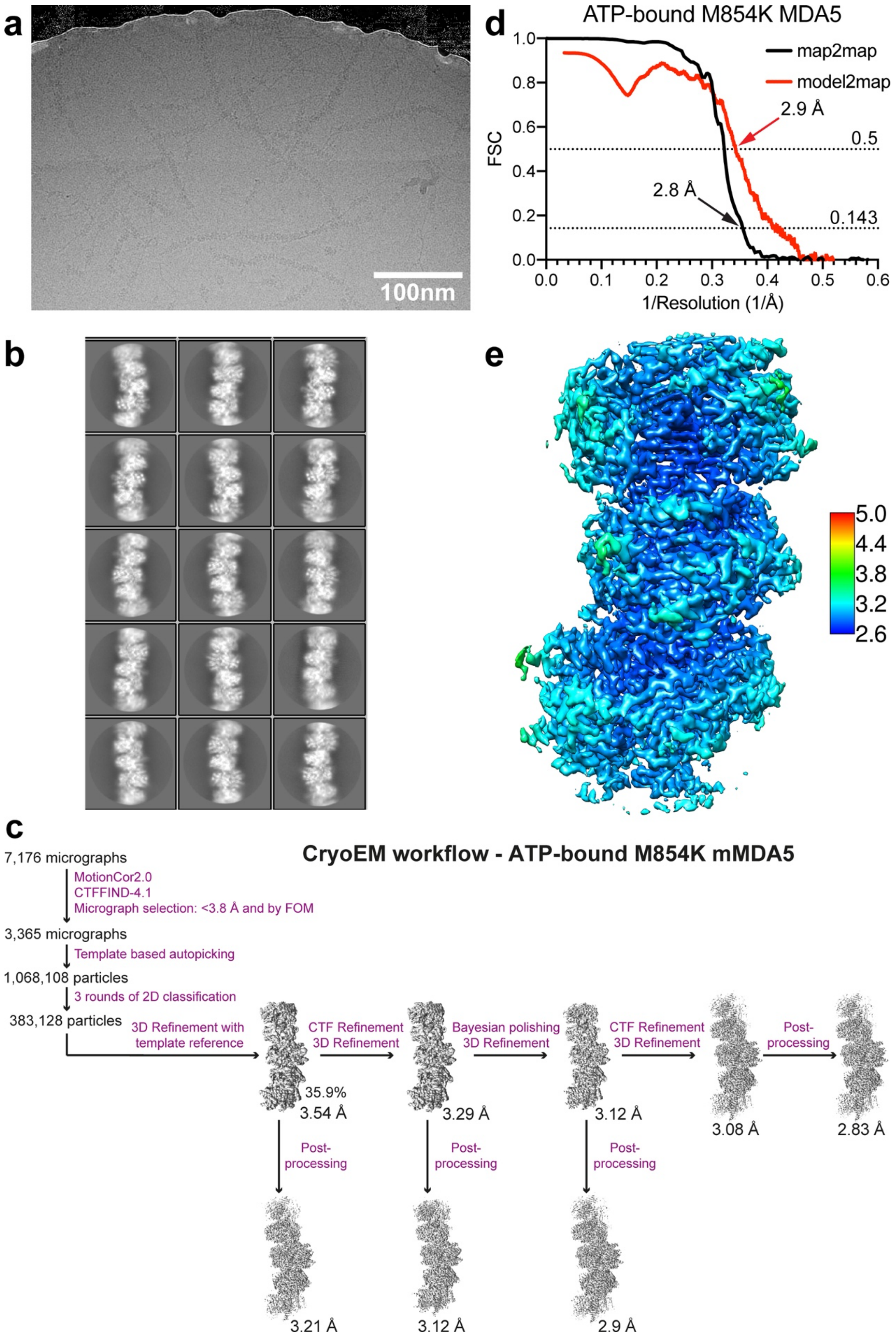

Supplementary Fig. 3. CryoEM reconstruction of M854K MDA5-dsRNA filaments with ATP bound. (**a**) A representative raw micrograph of ATP-bound M854K MDA5-dsRNA filaments. (**b**) Representative 2D classes of ATP-bound M854K MDA5-dsRNA filaments. (**c**) CryoEM image processing workflow. (**d**) Map-to-map Fourier Shell Correlation (FSC) and Map-to-model FSC of the ATP-bound M854K MDA-dsRNA filament reconstruction. (**e**) Local resolution of the final ATP-bound M854K MDA-dsRNA filament reconstruction.

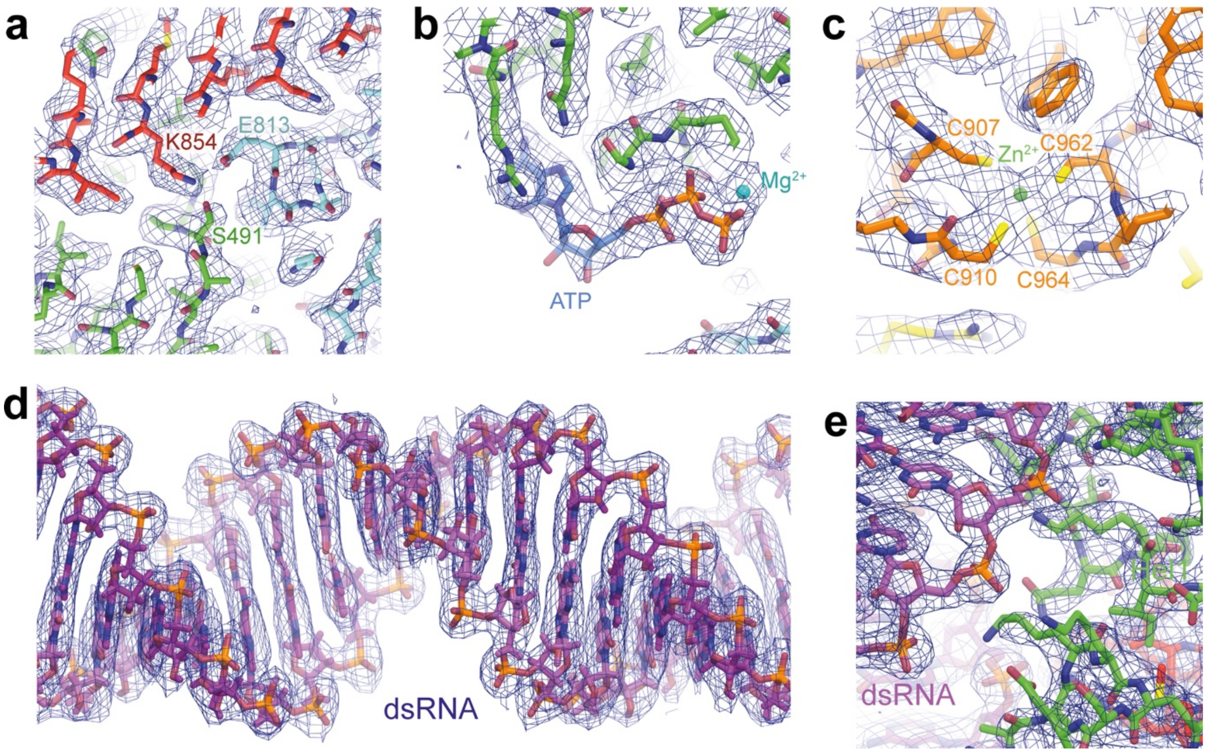

Supplementary Fig. 4. Samples of cryoEM density from the M854K MDA5-dsRNA filament structure. Representative samples of local cryoEM density from the reconstruction of M854K MDA5-dsRNA filaments with ATP bound with fitted and refined atomic models, drawn with PyMol v2.4.0 (Schrodinger, Ltd). (**a**) The region surrounding residue 854. Contour level, 3.8 σ. (**b**) The region surrounding residue the bound ATP molecule. Contour level, 3.8 σ. (**c**) The region surrounding the zinc ion in the C-terminal domain (CTD). Contour level, 4.5 σ. (**d**) The region surrounding the dsRNA. Contour level, 3.8 σ. (**e**) The dsRNA-Hel1 interface. Contour level, 5.8 σ.

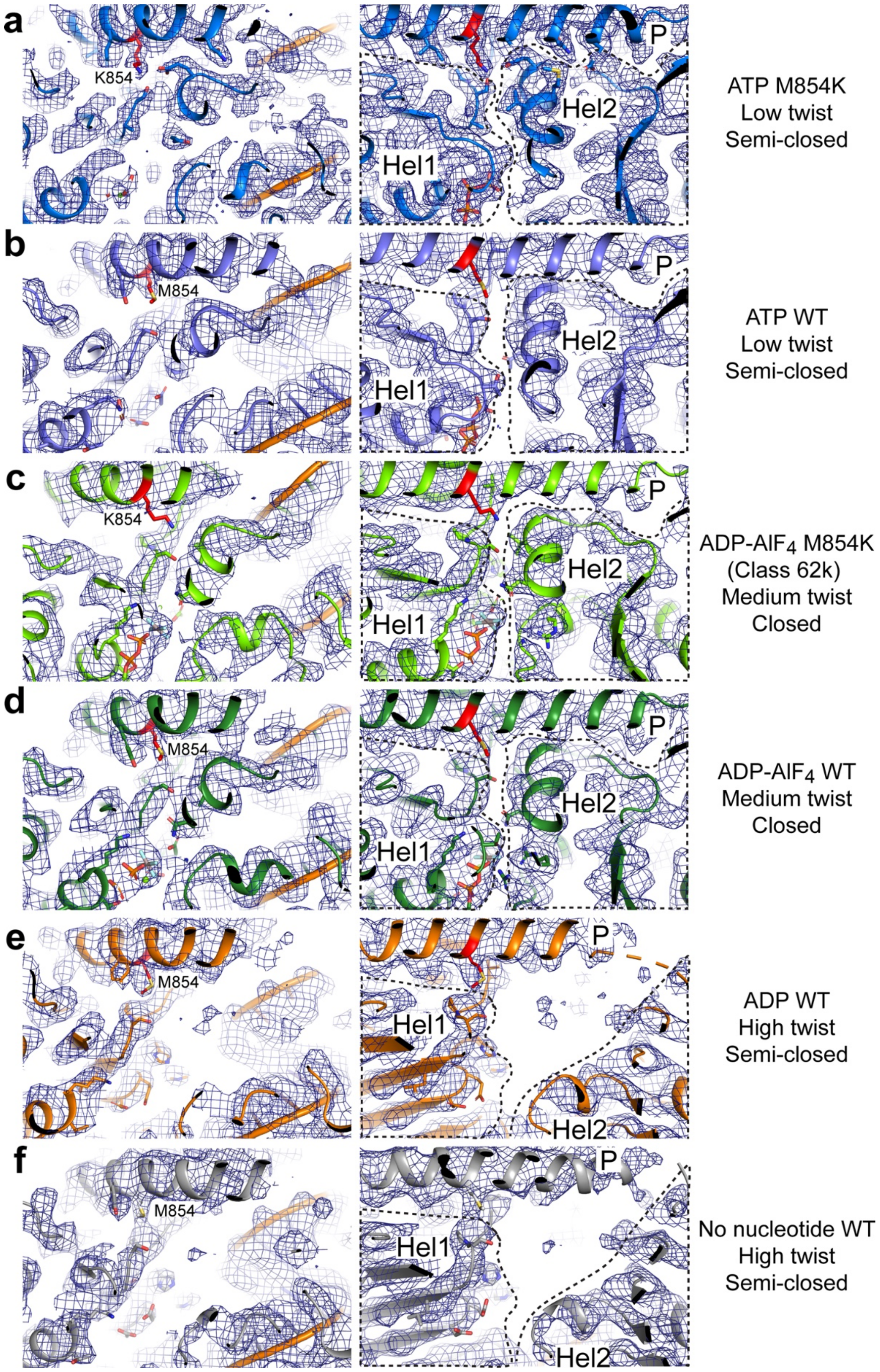

Supplementary Fig. 5. CryoEM density of MDA5-dsRNA filaments at different stages of ATP hydrolysis. Left, region around residue 854; right, Hel1-Hel2-pincer interface. 5.5-σ contour level in PyMol. (**a**) ATP-bound M854K MDA5 filament structure, 74˚ twist. (**b**) ATP-bound WT MDA5-dsRNA filament structure, 73˚twist (PDB:6GKM). (**c**) ADP-AlF_4-bound M854K MDA5-dsRNA filament structure, 88˚twist (Class 62k). (**d**) ADP-AlF_4-bound WT MDA5-dsRNA filament structure, 88˚twist (PDB:6GKH). (**e**) ADP-bound WT MDA5-dsRNA filament structure, 92˚ twist. (**f)** Nucleotide-free WT MDA5-dsRNA filament structure, 93˚ twist (PDB:6H66).

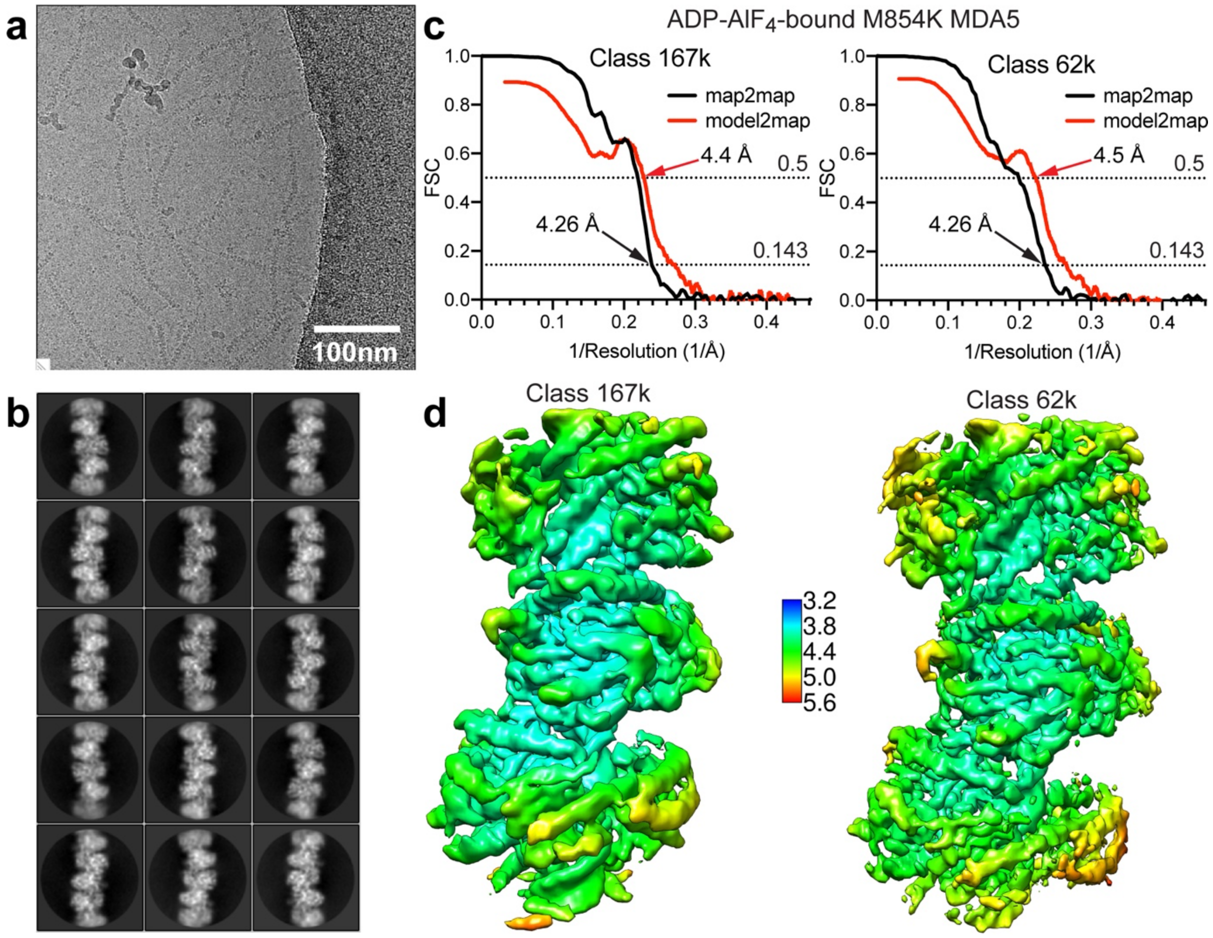

Supplementary Fig. 6. CryoEM reconstructions of ADP-AlF$_4$-bound M854K MDA5-dsRNA filaments. (**a**) A representative raw micrograph of ATP-AlF$_4$-bound M854K MDA5-dsRNA filaments. (**b**) Representative 2-D classes of ATP-AlF$_4$-bound M854K MDA5-dsRNA filaments. (**c**) Map-to-map Fourier Shell Correlation (FSC, red) and map-to-model FSC of ATP-AlF$_4$-bound M854K MDA5-dsRNA filament reconstructions (left, Class 167k; right, Class 62k). (**d**) Local resolution of the ATP-AlF$_4$-bound M854K MDA5-dsRNA reconstructions. Left, Class 167k; Right, Class 62k (see **Table 1**).

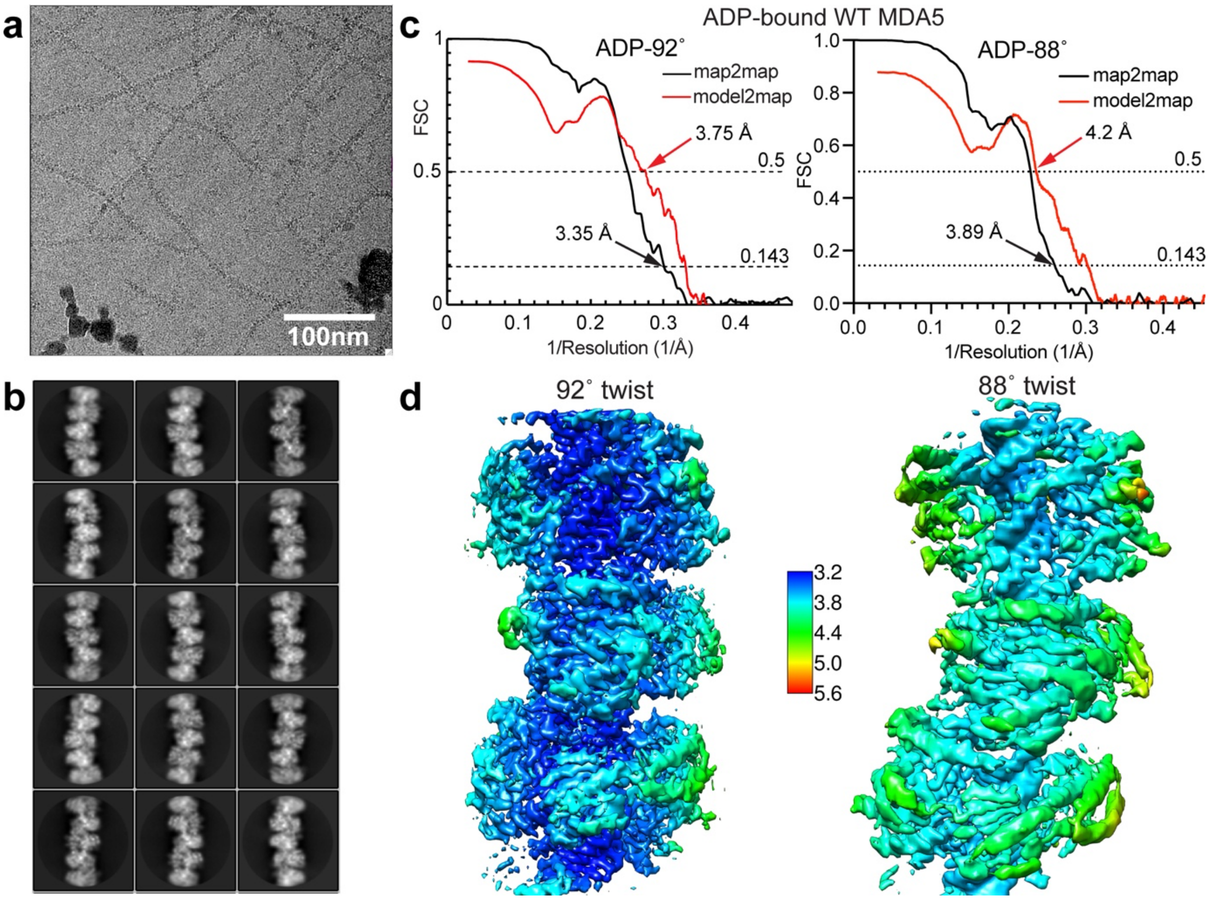

Supplementary Fig. 7. CryoEM reconstruction of WT MDA5-dsRNA filaments with ADP bound. (**a**) A representative raw micrograph of ADP-bound WT MDA5-dsRNA filaments. (**b**) Representative 2D classes of ADP-bound WT MDA5-dsRNA filaments. (**c**) Map-to-map Fourier Shell Correlation (FSC) and Map-to-model FSC of ADP-bound WT MDA5-dsRNA filament reconstructions. Left, 92˚ twist; right, 88˚ twist. (**d**) Local resolution of the ADP-bound WT MDA5-dsRNA filament reconstructions. Left, 92˚ twist; right, 88˚ twist (see **Table 1**).

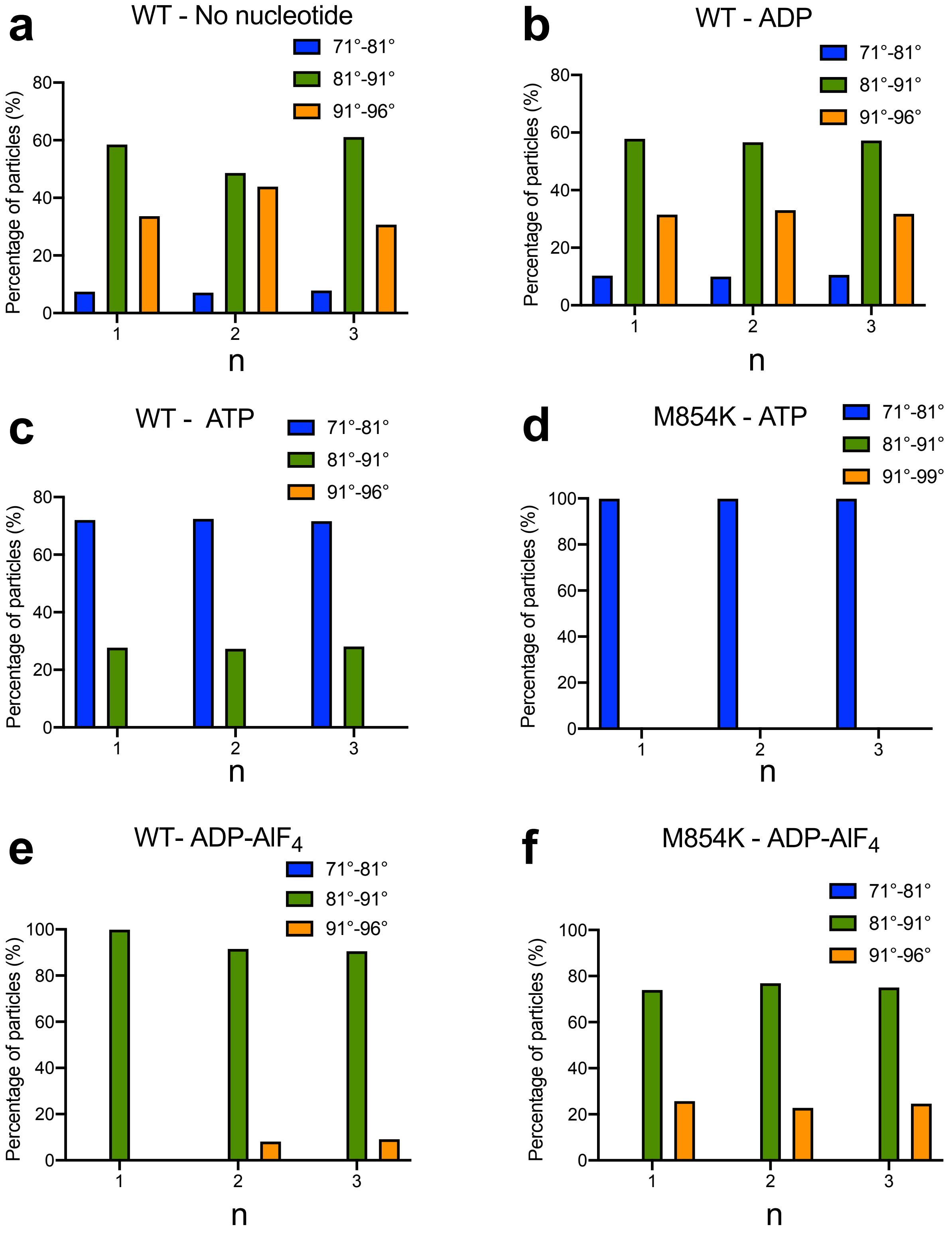

Supplementary Fig. 8. Reproducibility of the 3D classification of different MDA5 filaments samples. The percentage of segments from different MDA5 filaments datasets is plotted against twist distribution from three independent 3-D classification runs. Related to **Fig. 6**.
