# Supplementary material for: MDA5 disease variant M854K prevents ATP-dependent structural discrimination of viral and cellular RNA
Source: Nat Commun. 2021 Nov 18;12:6668. doi: 10.1038/s41467-021-27062-5 (PMC8602431; doi:10.1038/s41467-021-27062-5)
Supplement: Supplementary file 3 — Reporting Summary [file 41467_2021_27062_MOESM3_ESM.pdf]

nature portfolio

Corresponding author(s): Yorgo Modis

Last updated by author(s): Oct 7, 2021

Reporting Summary

Nature Portfolio wishes to improve the reproducibility of the work that we publish. This form provides structure for consistency and transparency in reporting. For further information on Nature Portfolio policies, see our Editorial Policies and the Editorial Policy Checklist.

Statistics

For all statistical analyses, confirm that the following items are present in the figure legend, table legend, main text, or Methods section.

n/a	Confirmed	
☐	☒	The exact sample size (*n*) for each experimental group/condition, given as a discrete number and unit of measurement
☐	☒	A statement on whether measurements were taken from distinct samples or whether the same sample was measured repeatedly
☐	☒	The statistical test(s) used AND whether they are one- or two-sided<br>*Only common tests should be described solely by name; describe more complex techniques in the Methods section.*
☒	☐	A description of all covariates tested
☒	☐	A description of any assumptions or corrections, such as tests of normality and adjustment for multiple comparisons
☐	☒	A full description of the statistical parameters including central tendency (e.g. means) or other basic estimates (e.g. regression coefficient) AND variation (e.g. standard deviation) or associated estimates of uncertainty (e.g. confidence intervals)
☐	☒	For null hypothesis testing, the test statistic (e.g. *F*, *t*, *r*) with confidence intervals, effect sizes, degrees of freedom and *P* value noted<br>*Give P values as exact values whenever suitable.*
☒	☐	For Bayesian analysis, information on the choice of priors and Markov chain Monte Carlo settings
☒	☐	For hierarchical and complex designs, identification of the appropriate level for tests and full reporting of outcomes
☒	☐	Estimates of effect sizes (e.g. Cohen's *d*, Pearson's *r*), indicating how they were calculated

Our web collection on statistics for biologists contains articles on many of the points above.

Software and code

Policy information about availability of computer code

Data collection	CryoEM data collection: EPU v.2.10
Data analysis	CryoEM image processing and reconstruction: RELION v3.1.3 (including its integrated implementation of MotionCor2), CtfFind4; atomic model building: COOT v.0.8.9.2, UCSF Chimera v. 1.14; atomic model refinement: PHENIX v1.18; scatter plots, histograms, error bars: GraphPad Prism 9.2.0, Microsoft Excel v. 16.52.

For manuscripts utilizing custom algorithms or software that are central to the research but not yet described in published literature, software must be made available to editors and reviewers. We strongly encourage code deposition in a community repository (e.g. GitHub). See the Nature Portfolio guidelines for submitting code & software for further information.

Data

Policy information about availability of data

All manuscripts must include a data availability statement. This statement should provide the following information, where applicable:

- Accession codes, unique identifiers, or web links for publicly available datasets
- A description of any restrictions on data availability
- For clinical datasets or third party data, please ensure that the statement adheres to our policy

The atomic coordinates were deposited in the Protein Data Bank with codes 7BKP, 7BKQ, 7NGA, 7NIC and 7NIQ. The cryoEM densities were deposited in the EM Data Bank with codes EMD-11937, EMD-12092, EMD-12213, EMD-12288 and EMD-12294. The raw electron micrographs were deposited in EMPIAR with codes 10630, 10653 and 10664. Full resolution original experimental images and source data used in the figures and supplemental figures are included in the supplementary information. Other data are available from the corresponding author upon reasonable request.

Field-specific reporting

Please select the one below that is the best fit for your research. If you are not sure, read the appropriate sections before making your selection.

- [x] Life sciences
- [ ] Behavioural & social sciences
- [ ] Ecological, evolutionary & environmental sciences

For a reference copy of the document with all sections, see nature.com/documents/nr-reporting-summary-flat.pdf

Life sciences study design

All studies must disclose on these points even when the disclosure is negative.

Sample size	No sample-size calculations were performed. Cell signaling assay, ATPase assays and 3-D classifications for twist distribution calculations were all performed in triplicate. This was sufficient to provide statistical significance wherever relevant.
Data exclusions	No data was excluded from the analyses in this study.
Replication	Results from all replicates were self-consistent. Some attempts at replication of ATPase assays with double or triple MDA5 mutants were unsuccessful due to instability of the protein after storage. ATPase assays were replicated in four different experiments. The WT and MDA5 proteins were each expressed and purified independently four times for this study. Cell signaling experiments were replicated in three different experiments.
Randomization	There were no human or animal participants in this study. Random allocation did not apply because samples were not subjected to co- or multivariate analysis.
Blinding	The investigators were not blinded to sample allocation because samples were all analyzed using the same quantitative measurements. Blinding to group allocation was not relevant because there were no human participants or control groups in the data collection for this study.

Reporting for specific materials, systems and methods

We require information from authors about some types of materials, experimental systems and methods used in many studies. Here, indicate whether each material, system or method listed is relevant to your study. If you are not sure if a list item applies to your research, read the appropriate section before selecting a response.

Materials & experimental systems

n/a	Involved in the study	
[ ]	[x]	Antibodies
[ ]	[x]	Eukaryotic cell lines
[x]	[ ]	Palaeontology and archaeology
[x]	[ ]	Animals and other organisms
[x]	[ ]	Human research participants
[x]	[ ]	Clinical data
[x]	[ ]	Dual use research of concern

Methods

n/a	Involved in the study	
[x]	[ ]	ChIP-seq
[x]	[ ]	Flow cytometry
[x]	[ ]	MRI-based neuroimaging

Antibodies

Antibodies used	Anti-FLAG primary antibody: Sigma-Aldrich, cat. no. F1804, RRID: AB_262044<br>Anti-actin primary antibody AC-40: Abcam, cat no. ab11003, RRID: AB_297660<br>Anti-MDA5 antibody: Enzo Life Sciences, cat. no. ENZ-ABS299, RRID: AB_2893162
Validation	Anti-FLAG primary antibody: purified by affinity chromatography (affinity tag purification); two major bands with purity >90% when analyzed by microfluidic gel capillary electrophoresis; protein content 1.0-1.1 g/l by UV absorbance at 280 nm (E1% = 12.5). See https://www.sigmaaldrich.com/GB/en/product/sigma/f1804 for immunofluorescence image. See also https://scicrunch.org/resolver/AB_262044, doi: 10.1038/ncomms7253<br>Anti-actin primary antibody AC-40: purified form tissue culture supernatant; See https://www.abcam.com/actin-antibody-ac-40-ab11003.html for immunohistochemistry image. See also https://scicrunch.org/resolver/AB_297660, doi: 10.1016/j.celrep.2020.108235<br>Anti-MDA5 antibody: Affinity purified; See for https://www.enzolifesciences.com/ENZ-ABS299/mda5-polyclonal-antibody/ immunohistochemistry, immunofluorescence and Western blot images. See also https://scicrunch.org/resolver/AB_2893162, doi: 10.1073/pnas.022637199

Eukaryotic cell lines

Policy information about cell lines

Cell line source(s)	Wild-type and ADAR1-knockout (KO) HEK293T cell lines were a kind gift from Charles Rice (The Rockefeller University). The wild-type HEK293T cells were originally sourced from ATCC (https://www.atcc.org) and the ADAR1 KO cells were generated in the Rice laboratory.
Authentication	None of the cell lines were reauthenticated. The ADAR1 KO cells were confirmed by Western blot to be negative for ADAR1 (this study).
Mycoplasma contamination	Cell lines were routinely tested for mycoplasma contamination using the MycoAlert detection kit (Lonza). All tests were negative.
Commonly misidentified lines (See ICLAC register)	No commonly misidentified cell lines were used.
